# USP10 Contributes to Colon Carcinogenesis via mTOR/S6K Mediated HIF-1α but Not HIF-2α Protein Synthesis

**DOI:** 10.3390/cells12121585

**Published:** 2023-06-08

**Authors:** Kateryna Kubaichuk, Thomas Kietzmann

**Affiliations:** Faculty of Biochemistry and Molecular Biology, University of Oulu, 90570 Oulu, Finland; thomas.kietzmann@oulu.fi

**Keywords:** proteasomal degradation, deubiquitinase, hypoxia, HIF-1a, colon cancer

## Abstract

Colorectal cancer ranks among the third most common human malignant diseases and is one of the leading causes of cancer-related deaths globally. Colon cancer cells are hypoxic and display disturbed protein homeostasis. Ubiquitin-ligase-initiated proteasomal degradation as well as its prevention by deubiquitinases (DUBs) are supposed to contribute to the above-mentioned disturbances. However, not much is known about the involvement of ubiquitinating and deubiquitinating enzymes in colon cancer and their effect on the hypoxia response. Here, we identify the DUB ubiquitin-specific protease 10 (USP10) as an important player in the control of colon cancer progression and a new modifier of the hypoxia response. Mechanistically, we show that knockout of USP10 in different colon cancer cells causes an elevation in HIF-1α but not HIF-2α protein levels under both normoxic and hypoxic conditions. In addition, the lack of USP10 increased cellular migration, reduced cell adhesion, and switched the energy phenotype towards increased glycolysis and enhanced extracellular acidification. These changes were at least partially caused by HIF-1α, as the knockdown of HIF-1α rescued the cellular phenotype caused by USP10 deficiency. Interestingly, the USP10-dependent increase in HIF-1 α was neither caused by enhanced transcription nor prolonged half-life but via mTOR/S6K mediated HIF-1α protein synthesis. Together, the current findings indicate that USP10 is able to participate in colon carcinogenesis by modulating the hypoxia response and may therefore represent a new therapeutic target.

## 1. Introduction

The last decade has shown that novel immune and chemotherapy regimens have improved cancer treatment substantially, although, surgery and chemotherapy are still the first treatment choices. Despite the progress, drug resistance often complicates chemotherapy. It is well established that chemotherapy resistance is associated with hypoxia and an abundance of hypoxia-inducible factors (HIFs) in a variety of human cancers, including pancreatic cancer, gastric cancer, neuroblastoma, non-small cell lung cancer (NSCL), and colorectal cancer (CRC) [1,2,3,4,5,6] suggesting that targeting the hypoxia response and HIFs might be a promising strategy in cancer therapy, especially in CRC.

HIFs exist as heterodimers composed of the hypoxia-inducible α-subunit (HIF-α) and the constitutively expressed β-subunit, also known as the aryl hydrocarbon receptor nuclear translocator (ARNT). So far, three major members of the HIF α-family have been described: HIF-1α, HIF-2α, and HIF-3α [7,8,9].

The abundance of HIF α-subunits under different oxygen tensions is mainly regulated on the post-translational level via hydroxylation followed by proteasomal degradation. Under normoxic conditions, HIF α-subunits are proline and asparagine hydroxylated [10]. This occurs due to the action of oxygen-dependent HIF-proline hydroxylases (PHD1-3/EglN1-3) and the asparagine hydroxylase factor inhibiting HIF (FIH). While the action of FIH attenuates the recruitment of cofactors such as CBP/p300, PHD action allows binding of the tumor suppressor protein von-Hippel–Lindau (VHL) that acts as the substrate recognition component of an E3 ubiquitin ligase complex. Subsequently, HIFs become ubiquitylated and undergo proteasomal degradation. Under hypoxic conditions the activities of the PHDs and FIH are reduced, hence allowing HIFs to escape their degradation, to recruit cofactors, and to undergo dimerization with ARNT to finally activate target genes. Importantly, hypoxia promotes feedback by activating PHD2 and PHD3 expression via HIFs. As a result, this leads again to rehydroxylation and degradation [11,12].

Apart from the oxygen-dependent degradation involving VHL, several signaling pathways control the ubiquitylation and proteasomal degradation of HIF α-subunits in an oxygen (i.e., hydroxylation)-independent manner by recruiting E3 ligases such as RACK1, HAF, MDM2, CHIP, Pellino3, and Fbw7 [13]. The action of E3s on HIF-1α can be opposed by several deubiquitinases (DUBs) such as ubiquitin-specific protease-7 (USP7), USP19, USP28, USP52, and OTUD7B [14].

During the last ten years, DUBs emerged as attractive therapeutic targets in cancer. About 100 DUBs are encoded by the human genome [15] and in addition to HIFs, a variety of DUBs are involved in the control of proliferation, apoptosis, DNA damage response, and cancer metastasis [15,16]. Considering that HIFα degradation in hypoxic cancers would be of benefit, it would be of advantage to use DUB inhibitors to shift the cellular balance towards those E3 ligases that promote HIFα degradation. However, as with PHDs, it may well be that feedback exists where hypoxia itself affects DUBs or where DUB inhibition affects the hypoxia response; both would complicate the use of DUB inhibitors in cancer therapy. However, currently, not much is known about such feedback mechanisms between hypoxia and DUBs. To gain an understanding, we screened whether hypoxia affects the expression of a panel of DUBs that are known to be associated with CRC. We found that the expression of the DUB ubiquitin-specific protease 10 (USP10) is reduced by hypoxia. Vice versa, we found that a lack of USP10 in different colon cancer cells promoted translation of HIF-1α via mTOR/S6K signaling and at the same time tumorigenesis. Together, our study provides evidence for the existence of a feedback cycle between hypoxia and DUBs at the level of USP10 and HIF-1α that contributes to colon carcinogenesis.

## 2. Materials and Methods

Chemicals. All biochemicals were of analytical grade and were obtained from commercial suppliers. 5,6-Dichloro-1-β-D-ribofuranosylbenzimidazole (DRB) and cycloheximide were purchased from Sigma-Aldrich and rapamycin from Calbiochem (Darmstadt, Germany). All substances were dissolved in DMSO (Sigma-Aldrich, Helsinki, Finland). Cells were treated with 100 µM DRB or 25 µg/mL cycloheximide.

Cell culture and maintenance. Human embryonic kidney cell line HEK293, mouse embryonic fibroblasts (MEFs), human colorectal carcinoma HCT116 cells were grown in Dulbecco’s modified Eagle’s medium (DMEM) (Biowest, Helsinki, Finland) and COLO320 cells were grown in Roswell Park Memorial Institute medium 1640 (RPMI 1640) (Sigma-Aldrich, Helsinki, Finland) supplemented with 10% fetal bovine serum (FBS) (Biochrom AG, Berlin, Germany), 1% non-essential amino acids (Sigma-Aldrich, Helsinki, Finland), and 0.5% ciprofloxacin hydrochloride (MP Biomedicals, Illkirch, France). Cells were maintained in 75 cm^2^ culture flasks at 37 °C and 97% humidity under normoxia (16% O_2_, 5% CO_2_, and 79% N_2_ (*v*/*v*)) or when indicated under hypoxia (5% or 1% O_2_, 5% CO_2_, and 90% N_2_ (*v*/*v*)) at 37°.

CRISPR/Cas9-mediated USP10 gene editing. To obtain a cell line lacking USP10, the clustered regulatory interspaced short palindromic repeats CRISPR/Cas9 nuclease approach was used. Custom short guide sgRNAs for each target, as well as the genotyping primers were designed in silico via the MIT CRISPR Design tool. Target sequences, allowing the introduction of two double-strand breaks in the USP10 gene were chosen and referred to as sgRNA-USP10-E4. A missense sequence (targeting a scrambled sequence OG from OriGene) was chosen as corresponding negative controls. The sgRNAs were combined with pSpCas9(BB)-2A-GFP (PX458) (Addgene). HCT116 and COLO320 cells were transfected with pSpCas9(BB)-2A-GFP-sgRNA-USP10-E4 and pSpCas9(BB)-2A-GFP-sgRNA-OG using the GeneJuice reagent (Novagen, Darmstadt, Germany) according to the protocol and sent for fluorescence-activated cell sorting (FACS) of clonal single cells.

Transient transfection of cells. HCT116 cells were transfected with the expression vectors for siRNA against HIF-1α (siSTRIKE-U6-HIF-1α) with the use of JetOptimus reagent (Polyplus, Illkirch, France) according to the manufacturer´s description. HEK293 cells were transfected with pSpCas9(BB)-2A-GFP-sgRNA-USP10-E4, pSpCas9(BB)-2A-GFP-sgRNA-OG, pLKO.1-Scr, pLKO.1-USP10-shRNA-1, pLKO.1-USP10-shRNA-2, and pDEST-HA-Flag-USP10 (Addgene) by the calcium phosphate co-precipitation method as described [17].

Western blot analysis. For the preparation of protein samples, all steps were performed on ice or at 4 °C. The cells were dissolved in 150 µL lysis buffer/60 mm cell culture dish (0.5 M EGTA, 0.5 M EDTA, 125 mM Na4P_2_O_7_, 10% Triton in Tris-buffered saline (TBS), 1 tablet of complete mini protease inhibitor (Roche, Mannheim, Germany)/10 mL)), harvested using a cell scraper and transferred to a 1.5 mL reaction tube. The analytical separation of proteins depending on their molecular weight was carried out in discontinuous polyacrylamide gels according to Laemmli [18]. Electrophoretic separation of the proteins was followed by transfer to a nitrocellulose membrane (GE Healthcare, Helsinki, Finland) using the semi-dry method. For detection of the proteins, the enhanced chemiluminescence (ECL) reaction was used. The membranes were incubated for 60 min in 1× PBS-T buffer with 5% fat-free milk to block non-specific binding sites, followed by incubation with the primary antibody at 4 °C overnight. The primary antibodies against USP10 (Bethyl Laboratories, #A300-900A, 1:1000), USP7 (Bethyl Laboratories, #A300-033A), YOD1 (Abgent, #AP11304b-ev), JOSD1 (Sigma-Aldrich, HPA001168), p-pS6K (Thr389) (Cell Signaling, #9205, 1:1000), pS6K (Cell Signaling, #9202, 1:1000), p-mTOR (Ser2448) (Santa Cruz, sc-293133, 1:500), mTOR (Santa Cruz, sc-517464, 1:500), p-4E-BP 1/2/3 (Santa Cruz, sc-271947, 1:500), 4E-BP1 (Santa Cruz, sc-9977, 1:500), VEGF (Santa Cruz, sc-152, 1:500), Glut1 (Santa Cruz, sc-377228, 1:500), HIF-1α (BD Bioscience, #610958, 1:500), HIF-2α (Novus, NB100-122; 1:500), α-tubulin (Sigma-Aldrich, T5168, 1:2000). Secondary anti-mouse (BioRad, 170-6516, 1:5000) and anti-rabbit (BioRad, 170-6515, 1:5000) HRP-conjugated antibodies were used after. The ECL Western Blotting Detection Reagent (Amersham, GE Healthcare, Helsinki, Finland) was used to detect the antibody-labeled proteins on X-ray films (Amersham, GE Healthcare, Helsinki, Finland), which were visualized by a short incubation in developer (1:500, Kodak, Chalon, France), followed by a washing step in H_2_O and an incubation in fixing solution (1:500, Kodak, Chalon, France). Films were scanned and analyzed using ImageJ software for quantification.

Luciferase assay. The 7.5 × 10^5^ HCT116 or COLO320 cells were plated onto 6 cm dishes and transfected with 2 mg of the HIF-responsive pGL3-Epo-HRE-Luc plasmid and 0.25 mg of Renilla luciferase reporter plasmid pRL-SV40 (Promega, Heidelberg, Germany). Media was replaced with fresh medium 12 h post-transfection and the cells were incubated under normoxic conditions or under 5% O_2_ at 37 °C for 18 h. Cells were lysed and the detection of luciferase activity in the lysate was performed with the Dual- Luciferase™ Reporter Gene Assay Kit (Berthold, Pforzheim, Germany) in the AutoLumat LB953 luminometer (Berthold, Pforzheim).

Quantitative real-time PCR. Total RNA was isolated with the help of GeneMATRIX Universal RNA Purification Kit (EURx) and GeneElute Mammalian Total RNA Miniprep Kit (Sigma) and used for reverse transcription according to the manufacturer’s instructions (qScript cDNA Synthesis Kit, Quantabio). qRT-PCR primers were designed in silico using Primer3Plus program. All reactions were performed in AB 7500 Real-Time PCR System using iTaq Universal SYBR Green Supermix reagent according to the manufacturer’s instructions (Bio-Rad). For each sample, expression of Hprt was used to normalize the amount of the investigated transcripts.

Cell proliferation assays. In order to assess proliferation via cell count, 4 × 10^5^ of cells were seeded onto 10 cm cell culture dishes and the cell number determination was performed every 24 h during 4 days of cell growth with the help of an automated cell counter (Countess II FL Automated Cell Counter, Invitrogen). For the real-time quantitative cell proliferation analysis, the cells were seeded onto 96-well plate (3 × 10^3^ or 5 × 10^3^ cells per well). Plates were moved into either the IncuCyte ZOOM System or into the IncyCyte S3 System (Essen BioScience, Newarc Close, UK) for the live phase contrast recording of cell confluence for at least 72 h with 3 h intervals. Where indicated, cells were starved in 3% FBS-containing media overnight before proceeding with the proliferation assay, and later repeatedly treated with 100 ng/mL EGF every 24 h. The confluence analysis was performed using the basic IncuCyte 2021C software settings.

Migration assay. Cell migration assay was performed using 24-well cell culture inserts with 8 µm pores (Becton Dickinson, Sigma-Aldrich, Helsinki, Finland). For this assay, cells were starved for 24 h in serum-free medium prior to the experiment. Then, 1 × 10^4^ cells were seeded on the upper wells of the 24-well chambers, while the lower wells are filled with medium containing 10% FBS. After incubation for 24 h, cells that migrated out onto the lower surface of membranes were fixed in 4% paraformaldehyde, stained with 0.0075% crystal violet solution (Applichem/VWR, Helsinki, Finland), and counted with ImageJ software.

Adhesion assay. To determine the adhesion ability of cells to a surface, 2 × 10^6^ cells per well were seeded onto 6-well plates. After 24 h one plate was transferred to an orbital shaker for 2 h at 250 rpm and 37 °C while the control plate remained in the incubator. The media was aspirated from the cells followed by three washing steps in 1× PBS and fixation in 1 mL 4% paraformaldehyde and staining with 0.0075% crystal violet solution (Applichem/VWR, Helsinki, Finland). Plates were scanned and analyzed using the ImageJ software for quantification.

Seahorse XFp analysis. The mitochondrial function in HCT116 cells was assessed via real-time measurement of O_2_ consumption rate (OCR) with the help of the XFp Extracellular Flux Analyzer (Seahorse, Agilent Technologies, Santa Clara, CA, USA) and Seahorse XFp Cell Mito Stress Tests according to the manufacturer’s protocol. The cells were seeded onto special cell culture miniplates in Seahorse XF base medium supplemented with 10 mM glucose, 1 mM sodium pyruvate, and 2 mM L-glutamine. Twelve hours after, the mitochondrial stress test was performed in response to oligomycin A (1 mM), FCCP (1 mM), and rotenone/antimycin A (0.5 mM). The glycolytic function of HCT116 cells was measured with the help of Seahorse Glycolysis Stress Test. The cells were plated as mentioned above and grown in Seahorse XF base medium supplemented with 2 mM L-glutamine. The glycolysis stress test was performed in response to 10 mM glucose, 1 mM oligomycin, and 50 mM 2-deoxyglucose. Obtained data were analyzed with the Seahorse XF Report Generator software (Wave, Agilent, 2.6.3.5, Santa Clara, CA, USA).

Statistical analyses. Within the whole study, all values are expressed as mean ± standard deviation (SD), whereas statistical significance was determined using the two-tailed Student’s *t* test.

## 3. Results

### 3.1. Hypoxia Reduces USP10 mRNA and Protein Levels

It has been reported that several DUBs—USP7, USP10, USP33, USP40, USP45, USP46, YOD1, and JOSD1 are involved in carcinogenesis [19,20,21,22,23,24,25,26,27,28,29,30]. Moreover, studies have shown that USP7, USP10, USP33, and USP46 are all involved in the progression of colorectal cancer [22,26,30,31,32,33].

To gain a first insight into whether hypoxia is able to influence the expression of DUBs involved in the overall survival of colon cancer (Supplementary Figure S1) we examined the mRNA and protein expression of USP7, USP10, USP33, USP40, USP45, USP46, YOD1, and JOSD1 in HEK293 cells exposed to normoxia (16% O_2_) and hypoxia (1% O_2_).

**Figure 1:**
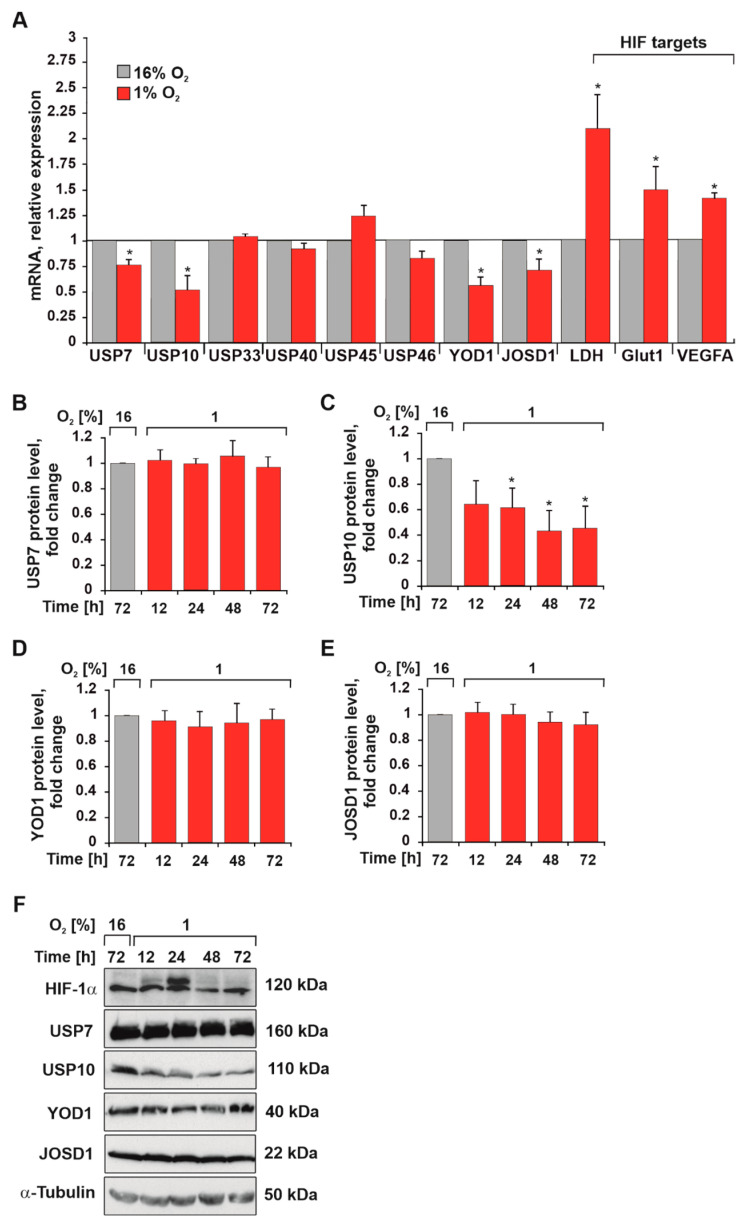
Hypoxia downregulates USP10. (**A**) Quantification of USP10, USP7, USP33, USP40, USP45, USP46, YOD1, JOSD1, LDH, GLUT1, and VEGFA mRNA levels in HEK293 cells cultured under normoxia (16% O_2_) and hypoxia (1% O_2_) for 9 h. HPRT was used to normalize the amount of the investigated transcripts. Analysis of USP7 (**B**), USP10 (**C**), YOD1 (**D**), and JOSD1 (**E**) protein levels. The DUBs levels in HEK293 cells under normoxia were set to 1. The values represent mean ± SD of 3 independent experiments: * significant difference (*p* < 0.05). (**F**) Representative USP7, USP10, YOD1, and JOSD1 immunoblots from HEK293 cells cultured under normoxia and hypoxia for 12, 24, 48, and 72 h. HIF-1α served as hypoxia control and α-tubulin as loading control.

**Figure 2:**
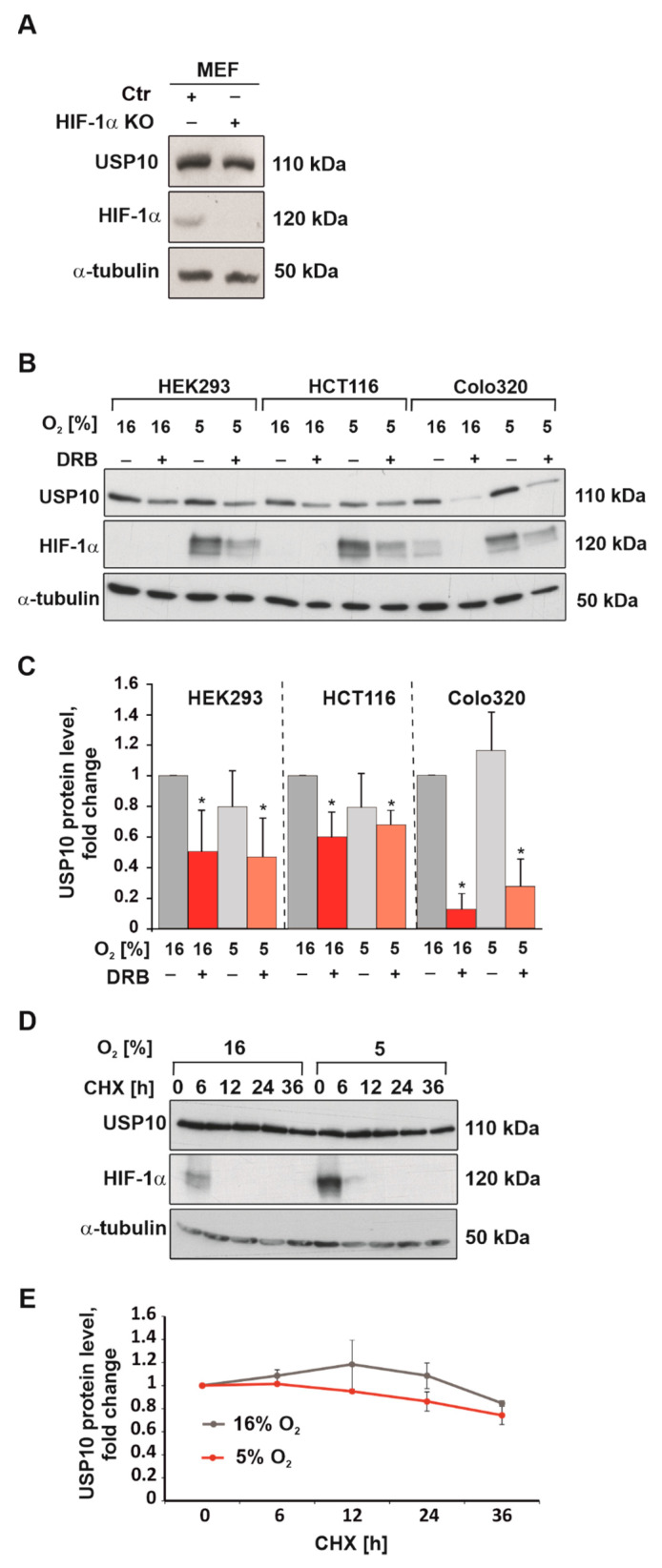
Hypoxia downregulates USP10 on transcriptional level. (**A**) Representative immunoblots of USP10 in MEFs with a HIF-1α knockout cultured under normoxia (16% O_2_). α-tubulin served as loading control. (**B**) Representative immunoblots of USP10 and c-MYC levels in the lysates from HEK293, HCT116, and Colo320 cells cultured under normoxia (16% O_2_) and hypoxia (5% O_2_) in the presence or absence of the transcriptional inhibitor DRB (100 µM) for 18 h. HIF-1α served as hypoxia control, and α-tubulin as loading control. (**C**) Analysis of USP10 protein levels. The USP10 level in each cell line under normoxia was set to 1. (**D**) Representative immunoblots of USP10 and HIF-1α levels in the lysates of HEK293 cells treated with cycloheximide (25 µg/mL) for 6, 12, 24, or 36 h. HIF-1α served as hypoxia control, and α-tubulin as loading control. (**E**) Analysis of USP10 protein levels. The USP10 levels under normoxia or hypoxia were set to 1. The values represent mean ± SD of 3 independent experiments: * significant difference (*p* < 0.05).

While hypoxia induced the mRNA levels of the HIF target genes (lactate dehydrogenase LDH, glucose transporter GLUT1, and vascular endothelial growth factor VEGF), the data show that mRNA levels of USP7, USP10, JOD1, and JOSD1 were reduced under hypoxia. The mRNA levels of USP33, USP40, USP45, and USP46 were not affected when cells were exposed to hypoxia (Figure 1A). Interestingly, Western blotting analyses showed that only USP10 protein levels were significantly downregulated by hypoxia in a time frame over 72 h (Figure 1B–F). Indeed, we observed an almost 60% reduction at both 48- and 72 h time points (Figure 1C,F). Further, the protein levels of USP10 were not affected by the lack of HIF-1α in mouse embryonic fibroblasts (MEF) under normoxia (Figure 2A), suggesting that the reduction in USP10 expression under hypoxia is not caused by the accumulation of HIF-1α. Together, these data indicated that from the DUBs investigated USP10 appears to be the most regulated by hypoxia. Therefore, our further analyses focused on the role of USP10 in the DUB-hypoxia feedback cycle.

To estimate the impact of transcription and translation on the changes in USP10 expression under hypoxia, cells were treated with the transcription inhibitor 5,6-Dichloro-1-β-D-ribofuranosylbenzimidazole (DRB) and the protein synthesis inhibitor cycloheximide (CHX). Treatment of cells with DRB could decrease the protein level of USP10 in HEK293 cells by 50% under both normoxia and hypoxia. A similar effect of DRB was observed in the human colon cancer cell lines HCT116 and COLO320. Hypoxia reduced the USP10 protein level by ~30% in HCT116 cells and ~70% in COLO320 cells (Figure 2B,C). On the other hand, cycloheximide did not affect USP10 protein levels under both normoxia and hypoxia (Figure 2D,E). This suggested that USP10 has a rather long half-life, and its downregulation under hypoxia is likely not caused by de novo protein synthesis failure. Together, the data from qRTPCR, DRB, and CHX experiments suggested that the downregulation of USP10 under hypoxia is due to its transcriptional inhibition.

### 3.2. USP10 Knockout Elevates HIF-1α but Not HIF-2α Protein Levels

**Figure 3:**
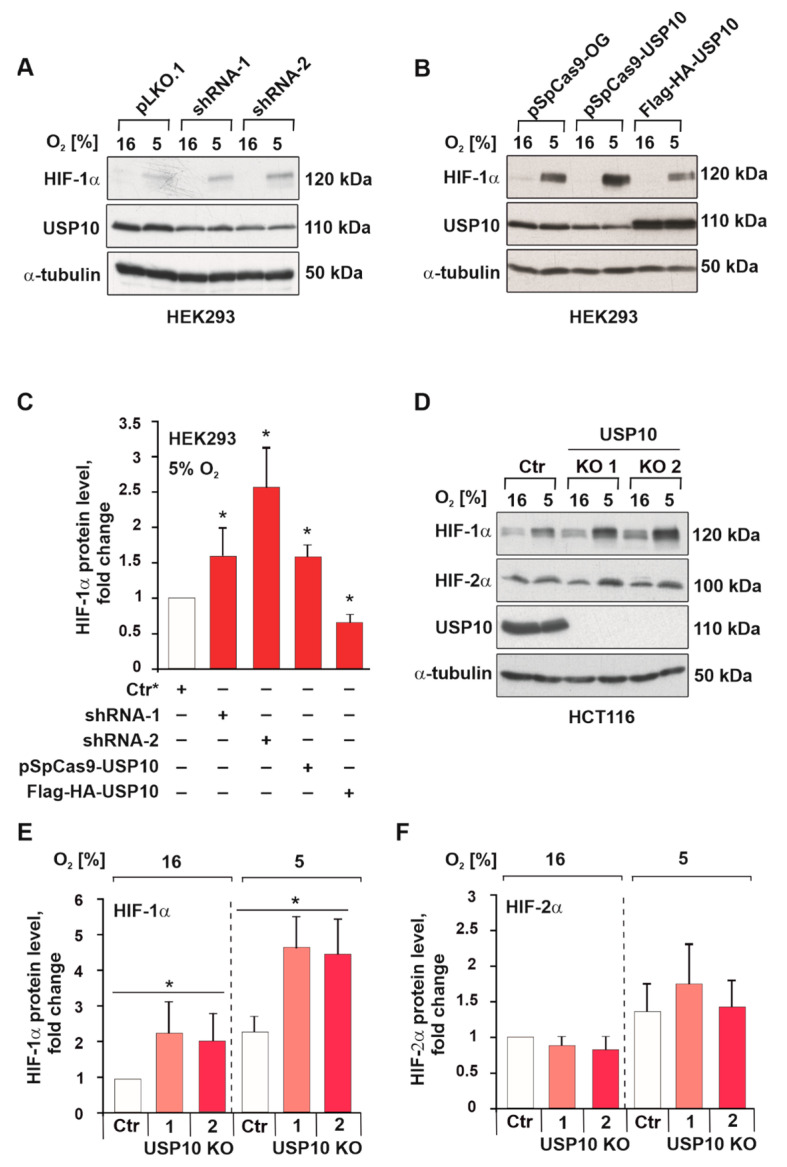
USP10 affects HIF-1α protein levels in HEK293 and HCT116 cells. (**A**) Representative immunoblots of USP10 and HIF-1α in the lysates of HEK293 cells expressing two independent shRNAs against USP10 (shRNA-1, shRNA-2) or a scrambled shRNA (pLKO.1). Cells were cultured under normoxic (16% O_2_) or hypoxic conditions (5% O_2_) for 5 h. α-tubulin served as loading control. (**B**) Representative immunoblot of USP10 and HIF-1α in cells transfected with pSpCas9-sgRNA-OG as control, pSpCas9-sgRNA-USP10-E4, and Flag-HA-USP10. Cells were cultured under normoxic (16% O_2_) or hypoxic conditions (5% O_2_) for 5 h. α-tubulin served as loading control. (**C**) Analysis of HIF-1α levels under hypoxia (5% O_2_). HIF-1α levels in cells expressing pLKO.1-Scr or pSpCas9-sgRNA-OG were set as 1. (**D**) Representative immunoblots of HIF-1α and HIF-2α levels in the lysates from HCT116 Ctr and USP10 KO1 and KO2 cells cultured under hypoxia for 5 h. α-tubulin served as loading control. (**E**) Analysis of HIF-1α protein levels. (**F**) Analysis of HIF-2α protein levels. The HIF levels in Ctr HCT116 cells were set as 1. The values are mean ± SD of 3 independent experiments: * significant difference (*p* < 0.05).

Considering that USP10 was downregulated by hypoxia, we next examined whether USP10 can exert a feedback effect on the hypoxia response by regulating HIF-1α and HIF-2α abundance. To do this, USP10 was first knocked down by two independent shRNAs. Interestingly, the knockdown of USP10 resulted in an increase in HIF-1α protein levels under hypoxia in HEK293 cells (Figure 3A,C). Similarly, transient knockdown of USP10 with the CRISPR/Cas9 sgRNA-USP10-E4 system resulted in similar increase in HIF-1α levels (Figure 3B,C). At the same time, USP10 overexpression caused a reduction in HIF-1α levels in HEK293 cells, confirming that USP10 is involved in the regulation of HIF-1α abundance (Figure 3B,C). Furthermore, USP10 knockout in HCT116 and COLO320 colon cancer cells led to higher HIF-1α protein levels under both normoxia and hypoxia. At the same time, no change was observed in HIF-2α protein levels upon knockout of USP10 in HCT116 cells, while in COLO320 HIF-2α was undetectable (Figure 3D–F, Supplementary Figure S2).

### 3.3. USP10 Deficiency Affects HIF-1α Signaling and Transcriptional Activity

**Figure 4:**
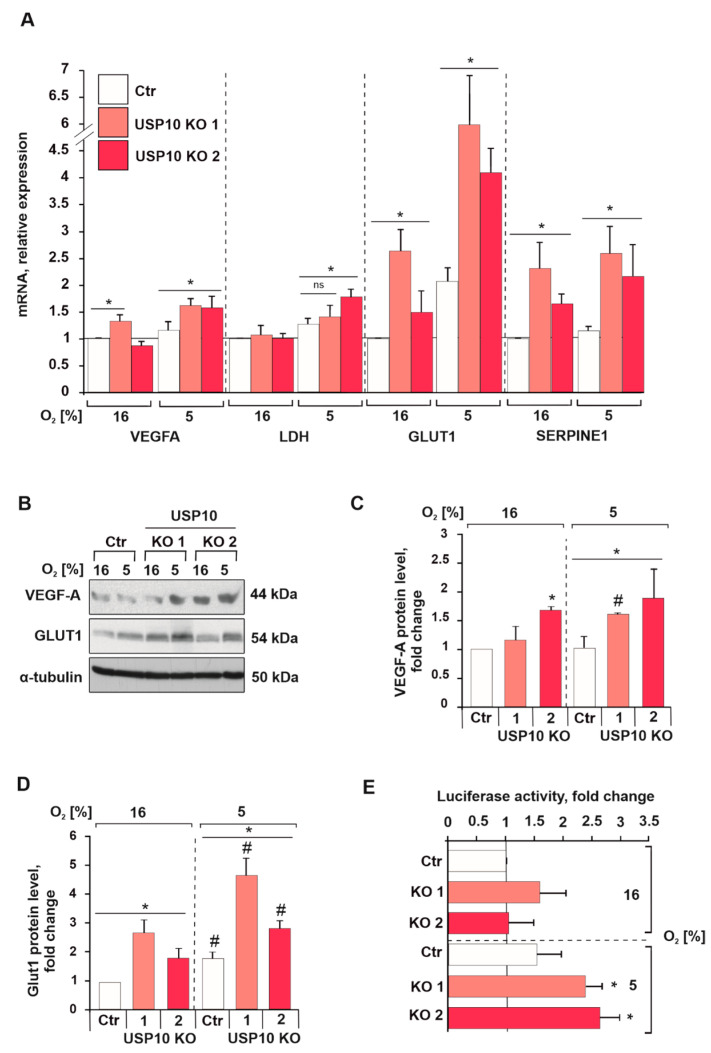
USP10 deficiency affects HIF-1α signaling and transcriptional activity. (**A**) Quantification of VEGFA, LDH, GLUT1, and SERPINE1 mRNA levels in Ctr and USP10 KO1 and KO2 cells. For each mRNA the levels in control cells were set to 1. HPRT was used to normalize the amount of the investigated transcripts. (**B**) Representative immunoblot of VEGF-A and GLUT1 protein levels in the lysates of HCT116 Ctr and USP10 KO1 and KO2 cells incubated in 5% O_2_ for 16 h. α-tubulin served as loading control; (**C**,**D**) analysis of VEGF-A and GLUT1 levels in HCT116 Ctr and USP10 KO1 and KO2 lysates. The VEGF-A and GLUT1 levels in normoxic HCT116 Ctr cells were set as 1. (**E**) USP10 knockout increases HIF transcriptional activity. HCT116 Ctr and USP10 KO1 and KO2 cells were transfected with a Luc reporter gene construct pGL3-HRE-Luc and treated with 5% O_2_ for 18 h. The Luciferase activity in Ctr cells at 16% O_2_ was set to 1. The values are mean ± SD of 3 independent experiments: * significant difference (*p* < 0.05) Ctr vs. USP10 KO1 or KO2 in either normoxia or hypoxia: # significant difference (*p* < 0.05) normoxia vs. hypoxia. ns: not significant.

Next, we investigated whether the USP10-dependent HIF-1α increases are of further functional relevance. Indeed, qRTPCR mRNA analyses showed that lack of USP10 had an additive effect on the hypoxia-dependent induction of VEGFA, LDH, GLUT1, and SERPINE1 mRNA levels (Figure 4A). In line, VEGF-A protein levels were increased in USP10 KO1 (40% increase) and KO2 (50% increase) cells compared to Ctr cells, while under normoxic conditions, only USP10 KO2 displayed significantly higher levels of VEGF-A protein compared to Ctr cells (Figure 4B,C). The protein level of GLUT1 was elevated in the USP10 KO1 and KO2 cells compared to Ctr under both normoxic (~3- and 2.5-fold increase for KO1 and KO2, respectively), and hypoxic conditions (~2.5- and 1.5-fold increase for KO1 and KO2, respectively) (Figure 4B,D). Additionally, luciferase reporter assays showed an increased HIF-1α transcriptional activity in both USP10 lacking cell lines (USP10 KO1 and KO2) upon exposure to hypoxia when compared to Ctr (Figure 4E). Similarly, COLO320 cells displayed an increase in HIF-1α transcriptional activity upon lack of USP10 under both normoxia and hypoxia (Supplementary Figure S2).

### 3.4. USP10 Deficiency Increases S6 Kinase Activity and HIF-1α Synthesis

**Figure 5:**
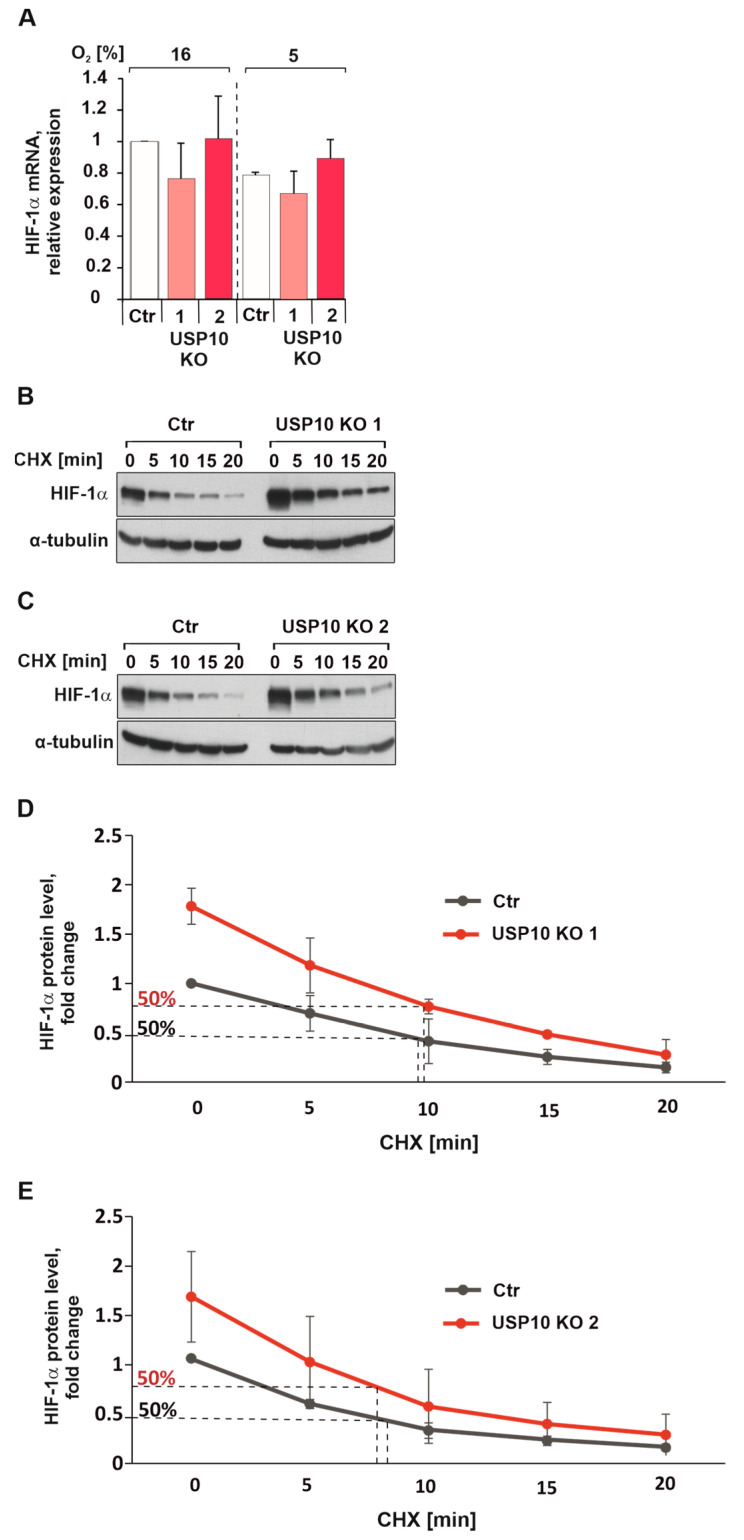
USP10 deficiency does not affect HIF-1α mRNA expression or protein half-life. (**A**) Quantification of HIF-1α mRNA levels in Ctr and USP10 KO1 and KO2 cells. HPRT was used to normalize the amount of the transcripts; (**B**,**C**) representative immunoblot of HIF-1α levels in the lysates from HCT116 Ctr and USP10 KO1 and KO2 cells, treated with translational inhibitor cycloheximide (CHX, 25 µg/mL) for 15, 30, and 60 min and incubated in hypoxia (5% O_2_) for 5 h. α-tubulin served as loading control; (**D**,**E**) Analysis of HIF-1α protein half-life. The HIF-1α levels in cycloheximide untreated Ctr HCT116 cells were set as 1.

It is known that the regulation of HIF-1α protein levels involves mainly stabilization of the protein but not the regulation of mRNA [34]. To exclude a possible effect of USP10 knockout on HIF-1α mRNA expression, qRTPCR was performed, and the obtained data show that neither USP10 deficiency nor hypoxia affected HIF-1α mRNA levels (Figure 5A). To test the hypothesis that USP10 regulates HIF-1α protein stability, HIF-1α half-life measurements were performed. Interestingly, the results show that USP10 knockout does not affect HIF-1α protein half-life (Figure 5B–E).

**Figure 6:**
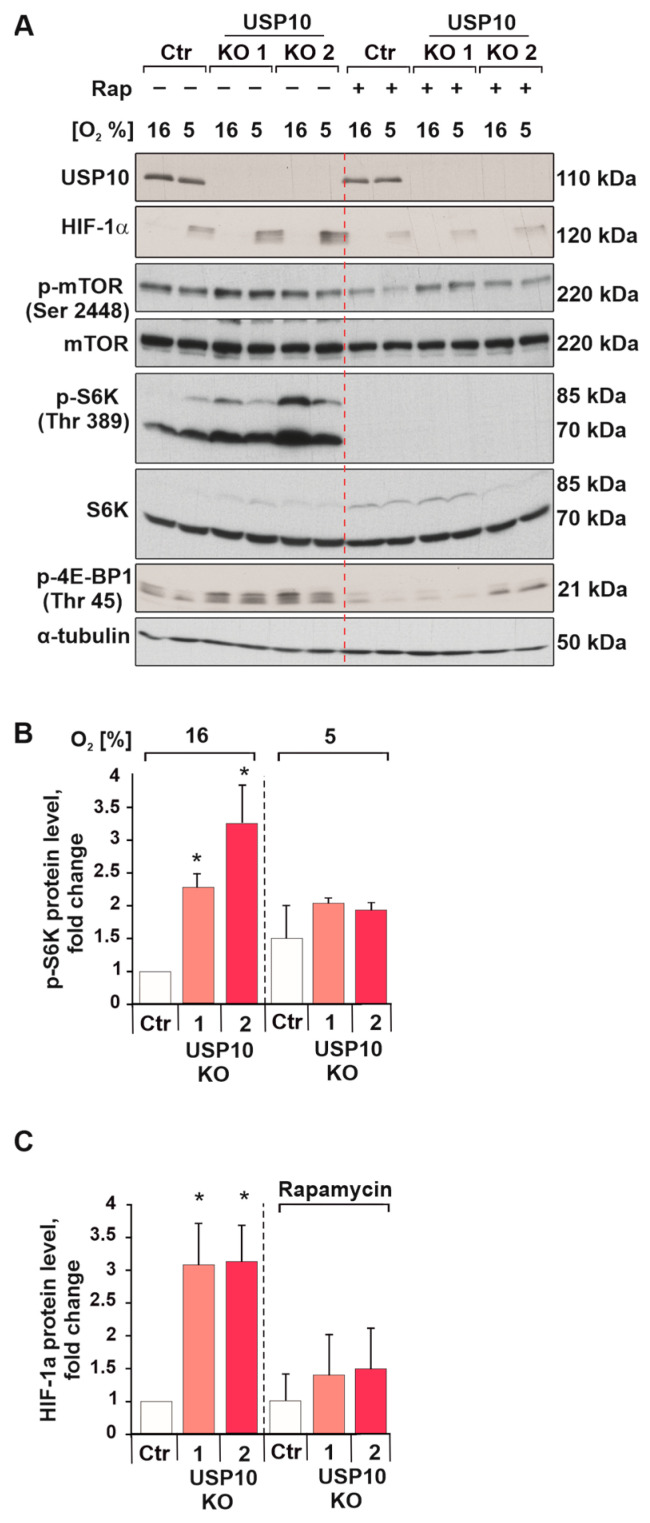
USP10 deficiency increases S6 kinase activity and HIF-1α synthesis. (**A**) Representative immunoblot of HIF-1α and p-S6 kinase levels in the lysates from HCT116 Ctr and USP10 KO1 and KO2 cells, pre-treated with mTOR inhibitor rapamycin (100 nM) for 16 h and exposed to hypoxia for 4 h. α-tubulin served as loading control. (**B**) Analysis of p-S6 kinase protein levels. (**C**) Analysis of HIF-1α protein levels. The p-S6K and HIF-1α levels in HCT116 Ctr cells were set to 1. The values are mean ± SD of 3 independent experiments: * significant difference (*p* < 0.05).

Another mechanism that regulates HIF-1α abundance is the control of HIF-1α protein synthesis. So far it is known that the mTOR pathway can affect HIF-1α translation [35]. To test whether the increase in HIF-1α protein levels in USP10 KO cells is caused by activation of mTOR, cells were treated with the mTOR inhibitor rapamycin (100 nM). While USP10-lacking cells displayed higher phosphorylation levels of mTOR and its downstream target S6 kinase compared to Ctr cells (Figure 6A–C), treatment with rapamycin inhibited activation of mTOR, S6 kinases and a translation repressor protein 4E-BP. At the same time, rapamycin caused a decrease in HIF-1α levels in USP10-lacking cells (Figure 6A,C). Thus, it can be assumed that higher HIF-1α abundance in USP10 KO cells is caused, at least partially, by mTOR/S6K pathway activation.

### 3.5. USP10 Knockout Affects the Cellular Energy Phenotype and Induces Glycolysis

**Figure 7:**
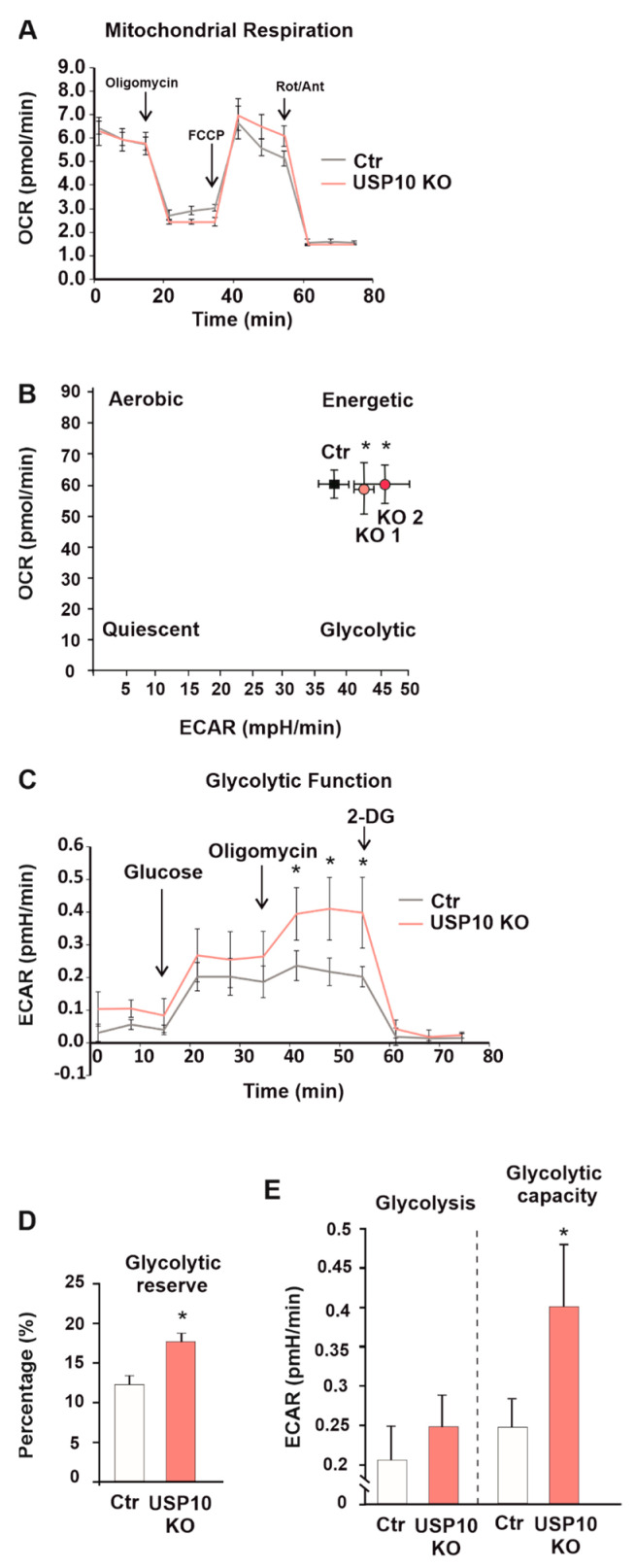
USP10 knockout affects the energy phenotype in HCT116 cells. (**A**,**C**) The oxygen consumption rate (OCR) (**A**) and extracellular acidification rate (ECAR) (**C**) of HCT116 Ctr and USP10 KO cells were measured under basal conditions and after the sequential addition of 1 mM oligomycin, 1 mM FCCP, and 0.5 mM rotenone/antimycin (**A**) or 10 mM glucose, 1 mM oligomycin and 50 mM 2-deoxyglucose (**C**). (**B**) The energy phenotypes of HCT116 Ctr and USP10 KO1 and KO2 cells. * Significant difference between extracellular acidification rate (ECAR) values of KO1 or KO2 vs. Ctr cells. (**D**) Analysis of glycolysis rate and glycolytic capacity in HCT116 Ctr and USP10 KO cells. (**E**) Analysis of glycolytic reserve in HCT116 Ctr and USP10 KO cells in percentages (%). * Significant difference between respective values of Ctr and USP10 KO cells. Data are mean ± SD of 3 independent experiments: * significant difference (*p* < 0.05).

Accumulation of HIF-1α in the cells predominantly correlates with the cells’ metabolic phenotype, as the cancer cells need to reprogram their metabolism, switching between glycolytic and aerobic phenotypes. To test whether the USP10 knockout-induced increase in HIF-1α was accompanied by energy phenotype changes, the Agilent Seahorse Mito Stress, Energy Phenotype, and Glycolysis Stress tests were used. One parameter in these assays is the oxygen consumption rate (OCR) which serves as an important indicator of normal cellular function. It is used as a parameter to study mitochondrial function as well as a marker of factors triggering the switch from healthy oxidative phosphorylation to aerobic glycolysis in cancer cells. The basal OCR in HCT116 Ctr cells was around 70  pmol/min/1500 cells, and no significant changes were found when comparing this to the OCR of USP10 knockout cells. As expected, the addition of the OXPHOS inhibitor oligomycin, decreased OCR levels below the basal line, suggesting an active oxidative phosphorylation concomitant to the observed glycolytic activity. Mitochondrial uncoupling with FCCP increased the oxygen consumption in both USP10 KO and Ctr cells, and inhibition of the mitochondrial electron transport chain with rotenone and antimycin, induced a strong OCR decrease. These responses were similar in USP10 knockout and Ctr HCT116 cells indicating that the absence of USP10 does not affect mitochondrial respiration in HCT116 cells (Figure 7A). Moreover, these analyses revealed that USP10 KO cells were characterized by a higher extracellular acidification (ECAR) level when compared to the Ctr cells which is indicative of a more pronounced glycolytic metabolism (Figure 7B).

Although the glycolysis stress test did not show an increased glycolysis rate (Figure 7C,E), glycolytic capacity, calculated as the difference between ECAR following the injection of 1 µm oligomycin, and the basal ECAR reading, was increased. The glycolytic reserve was also significantly increased in the USP10 KO cells (Figure 7D). Together, these results indicate that USP10 can act as an important regulator of cellular metabolism in HCT116 cells.

### 3.6. HIF Knockdown Rescues USP10-Dependent Migration and Adhesion but Not Proliferation

**Figure 8:**
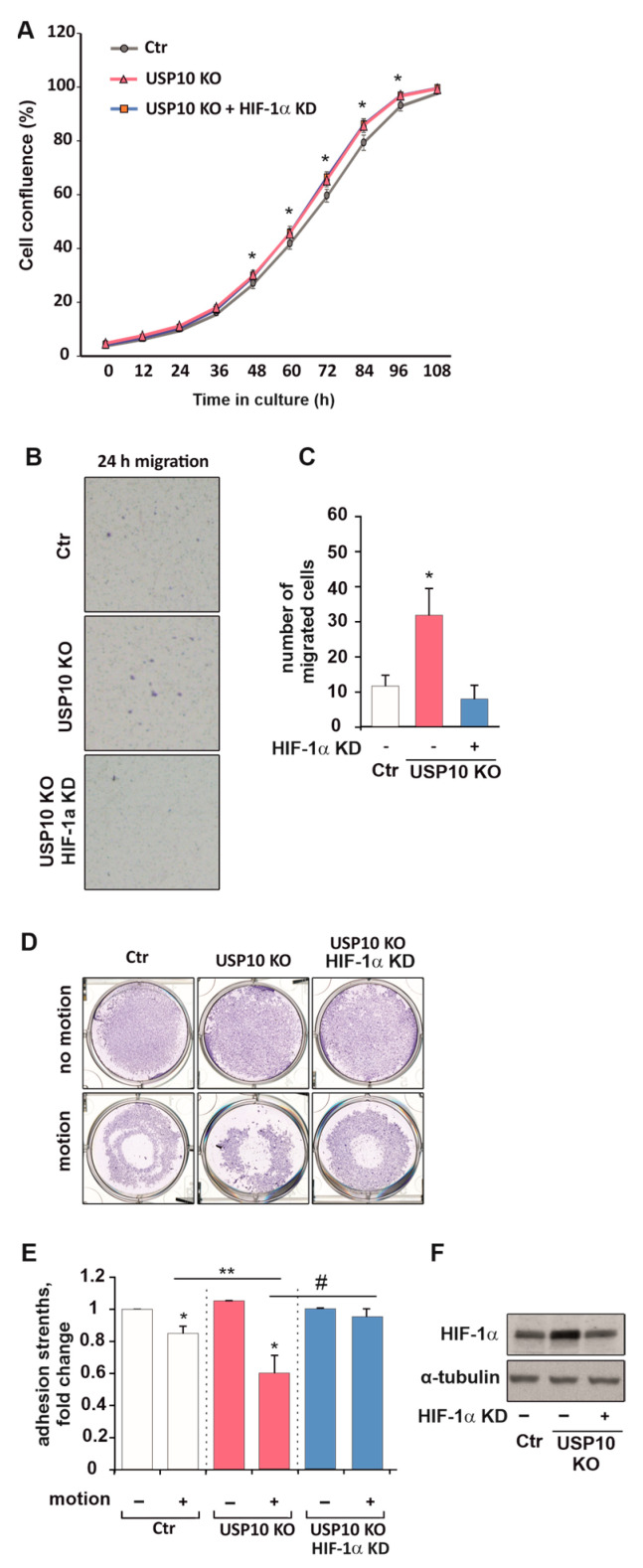
HIF knockdown rescues USP10-dependent migration and adhesion but not proliferation. (**A**) Live cell proliferation analysis of HCT116 control cells (Ctr), USP10 KO, and USP10 KO cells with a HIF-1α knockdown. * Significant difference (*p* < 0.05) between relative confluence values of USP10 KO or USP10 KO + HIF-1α knockdown vs. Ctr cells at each time point. (**B**) Representative images of the cell culture insert from the 24 h migration assay of HCT116 Ctr cells and USP10 KO cells. (**C**) Quantification of the Transwell migration assay towards serum. * Significant difference (*p* < 0.05) between USP10 KO vs. Ctr. (**D**) Representative images of the whole cell culture wells after adhesion assay. (**E**) Analysis of cellular adhesion strengths. Adhesion in the motionless samples was set to 1. * significant difference, USP10 KO cells vs. Ctr, ** significant difference between Ctr vs. USP10 KO in motionand # significant difference between USP10 KO vs. USP10 KO with HIF-1α knockdown in motion. (**F**) Representative immunoblot of HIF-1α in the lysates of HCT116 Ctr, USP10 KO, and USP10 + HIF-1α knockdown cells. α-tubulin served as loading control. Data are mean ± SD of 3 independent experiments: * significant difference (*p* < 0.05).

Increased proliferation, colony-forming ability, motility, and decreased adhesiveness of tumor cells are hallmarks of a carcinogenic process. Here, we show that USP10 knockout significantly increased proliferation in HCT116 cells, which was observed already after 48 h by a cell counting assay (Supplementary Figure S3). Next, we tested whether the increased level of HIF-1α in USP10 KO cells could be responsible for the higher proliferation of these cells compared to Ctr cells. To do this, we knocked down HIF-1α in the Ctr and USP10 KO cells. Interestingly, we found that the knockdown of HIF-1α in USP10 KO cells could not reverse their increased cellular proliferation. This finding suggests that HIF-1α is not primarily responsible for the enhanced proliferation of USP10 knockout cells (Figure 8A).

The USP10 knockout also induced migration, strengthened adhesion, and a change in epithelial-to-mesenchymal markers ZEB1 and E-Cadherin when compared to Ctr cells (Supplementary Figure S3). At the same time, the knockdown of HIF-1α in USP10 KO cells caused a three-fold decrease in cell migration and an almost 40% increase in adhesion strength (Figure 8B–E). These findings indicate that the USP10-mediated changes in HIF-1α are more linked to colon cancer cell migration rather than proliferation.

## 4. Discussion

The current study showed the existence of a feedback cycle between the hypoxia response and the amount of DUBs at least at the levels of USP10 and HIF-1α. In particular, the study revealed that USP10 is a hypoxia-regulated DUB that can contribute to HIF-1α protein synthesis via mTOR pathway regulation. Thereby, USP10 knockout affects the cellular energy phenotype and shifts it towards a more glycolytic metabolism, which goes in line with increased HIF-1α transcriptional activity. Further, USP10 knockout increases cellular migration and adhesiveness via activation of HIF-1α signaling especially in colon cancer cells.

USP10 recently gained interest due to its potential involvement in DNA repair that is linked to carcinogenesis. Further, USP10’s importance for life is underlined by the fact that USP10 knockout mice died within 1 year due to bone marrow failure with pancytopenia suggesting that USP10 controls differentiation, and apoptosis which are also crucially linked to tumorigenesis. Indeed, several malignancies like certain breast cancers and glioblastoma display prominent USP10 overexpression. By contrast, USP10 expression is downregulated in other malignancies including gastric carcinoma, lung, and colon cancers. Interestingly, USP10 was reported to have opposing roles in the same cancer. This is exemplified in colon cancer where USP10 seems to have tumor-promoting functions [28] or tumor-suppressive activities [31]. These phenomena suggest that the role of USP10 in cancer is rather unclear and may depend on the context and environmental settings such as hypoxia.

The presented study aimed to further discover the role of USP10, a member of the DUB family, especially in colon cancer cells. Investigating the role of USP10 is essential to partly clarify the specificity and activity of DUB family members to develop DUB-targeting drugs in future colon cancer therapies.

It was shown that loss of USP10 is accompanied by increases in HIF-1α levels and a changed energy phenotype of the cells that is characterized by an enhanced acidification rate and a shift towards increased glycolysis [36].

Although the main cellular mechanism to regulate HIF-1α protein abundance is via its stabilization due to hypoxia-mediated PHDs inhibition [34], cells display another mechanism to regulate HIF-1α abundance—HIF-1α protein synthesis [35]. So far, it is known that the mTOR signaling cascade can affect HIF-1α translation and stimulation of this pathway, for example by growth factors, hormones, or oncogenes/tumor suppressor mutations, which can lead to HIF-1α synthesis, even under normoxic conditions [37]. Indeed, USP10 knockout did not affect cellular HIF-1α protein longevity in HCT116 cells but induced activation of the PI3K/mTOR pathway. While the current finding suggests that USP10 affects HIF-1α protein abundance through its protein synthesis activation, we can at the moment not exclude whether this is a HIF-1α-specific phenomenon or would also affect other proteins.

There are numerous reports indicating that HIF-1α has inhibitory effects on cell proliferation, for instance by mediating cell cycle arrest through both non-transcriptional effects on the MCM helicase during DNA replication and through transcriptional activation of genes encoding the CDK inhibitors p21 and p27 [38]. In addition, HIF-1α can also counteract the effects of Myc on proliferation [39]. On the other hand, HIF-1α can activate cellular proliferation by inducing various growth factors in different cancer cells. In addition to peptide growth factors, HIF-1α also induces cell proliferation via activation of the ERK1/2 MAPK signaling pathway [40]. However, this study has shown that the changes in proliferation caused by USP10 knockout in HCT116 are not conducted via HIF-1α. Nevertheless, findings that USP10 knockout induces HIF-1α levels to fit with the enhanced proliferation data as well as with the changed energy phenotype [39].

An increase in intracellular HIF-1α is generally causing epithelial to mesenchymal transition (EMT) of the cells [41]. This leads to pronounced induction of cellular motility and migration. Indeed, high levels of HIF-1α in USP10 knockout HCT116 cells go well in line with increased migration and reduced adhesiveness of the cells. Moreover, these changes in cellular behavior are at least partially caused by HIF-1α [42], as the knockdown of HIF-1α is rescuing the cellular phenotype caused by USP10 deficiency.

Overall, the data support the view that feedback cycles between the hypoxia response and DUBs exist and that USP10 can participate in carcinogenesis by modulating the hypoxia response pathway and regulating several aspects that are important for cellular growth in colon cancer cells.

## Data Availability

Publicly available datasets were analyzed in this study (http://kmplot.com, accessed on 1 April 2023).The data presented in this study are available on request from the corresponding author.

## Supplementary Materials

The following supporting information can be downloaded at: https://www.mdpi.com/article/10.3390/cells12121585/s1, Figure S1. Kaplan-Meier overall survival analysis of 165 colorectal adenocarcinoma patients for USP7, USP10, USP33, USP40, USP45, USP46, YOD1 and JOSD1 RNASeq data. Data were generated with the online tool KM plotter (http://kmplot.com, accessed on 1 April 2023) [43]. Figure S2. USP10 affects HIF-1α protein level in COLO320 cells. (A) Representative immunoblot of USP10 and HIF-1α levels in the lysates of COLO320 Ctr cells and in USP10 KO1 and USP10 KO2 cells. Cells were cultured under normoxic (16% O_2_) or hypoxic conditions (5% O_2_) for 5 h. α-tubulin served as loading control. (B) Analysis of HIF-1α protein levels in Ctr COLO320 cells was set as 1 (C) COLO320 Ctr and USP10 KO1 and KO_2_ cells were transfected with a Luc reporter gene construct pGL3-EPO-HRE-Luc and treated with 5% O_2_ for 18 hours. The Luciferase activity of pGL3-EPO-HRE-Luc transfected Ctr at 16% O_2_ was set to 1. The values are mean ± SD of 3 independent experiments; * significant difference (*p* < 0.05). Figure S3. USP10 knockout increases the proliferation and motility and decrease adhesiveness in HCT116 cells. (A) Analysis of cell count assay of HCT116 control cells and USP10 lacking cells measured every 24, 48 and 72 h. (B) Representative images of the cell culture inserts from the 24 h migration assay of HCT116 Ctr cells and USP10 KO1 and KO_2_ cells. (C) Quantification of the Transwell migration assay towards serum. (D) Representative images of the whole cell culture wells after adhesion assay. (E) Analysis of cellular adhesion strengths. Adhesion of motionless samples was set to 1. (F) Representative immunoblot of E-Cadherin, α-SMA and ZEB1 levels in the lysates of HCT116 Ctr cells and in USP10 KO1 and USP10 KO2 cells. α-tubulin served as loading control. (G) Analysis of E-Cadherin, α-SMA and ZEB1 protein levels in Ctr HCT116 cells was set as 1 Data are mean ± SD of 3 independent experiments; * significant difference (*p* < 0.05) between USP10 KO cells vs. Ctr.
