# Supplementary material for: USP10 Contributes to Colon Carcinogenesis via mTOR/S6K Mediated HIF-1α but Not HIF-2α Protein Synthesis
Source: Cells. 2023 Jun 8;12(12):1585. doi: 10.3390/cells12121585 (PMC10297363; doi:10.3390/cells12121585)
Supplement: Supplementary file 1 [file cells-12-01585-s001.zip › cells-2398258-supplementary.pdf]

Supplementary Materials

qRTPCR primer list

Gene name	Forward primer (5´to 3´)	Reverse primer (5´to 3´)
USP10	GATGCACTGGAGAGCTTGGT	ATAAGCTTCTGGCACCCACC
USP7	GCAGATAGTCGCAGAGGACC	ACTCAGCAAGCGAGGAGTTC
USP33	CTCTGGTTGCCGTATTTGATGA	TCGAGCTAGTCCTCCACAATC
USP40	ATGAGGCAACATGATGTGCAG	TGGTACAGACGATAGATGAGGTC
USP45	ATGCGGGTGAAAGATCCAACT	CACGCTGATAGCATCACTTACA
USP46	CACTATTGCGGACATCCTTAG	CAGGTTCGTTCATGTTGCCA
YOD1	GGTCAGCGAATCCTCGTCG	CACCACGTTTAGTAAATGCAGGT
JOSD1	GGGATACGCTGCAAGAGATTT	CCATGACGTTAGTGAGGGCA
HIF1A	ATCCATGTGACCATGAGGAAATG	TCGGCTAGTTAGGGTACACTTC
EPAS1 (HIF-2a)	GGAGGTGTTCTATGAGCTGG	AGCTTGTGTGTTCGCAGGAA
LDH	TTGACCTACGTGGCTTGGAAG	GGTAACGGAATCGGGCTGAAT
SLC2A1 (GLUT1)	TCTGGCATCAACGCTGTCTTC	CGATACCGGAGCCAATGGT
VEGFA	AGCTGCGCTGATAGACATCC	CTACCTCCACCATGCCAAGT
Serpine1 (PAI-1)	AGCTCCTTGTACAGATGCCG	ACAACAGGAGGAGAAACCCA
HPRT	CCTGGCGTCGTGATTAGTGAT	AGACGTTCAGTCCTGTCCATAA

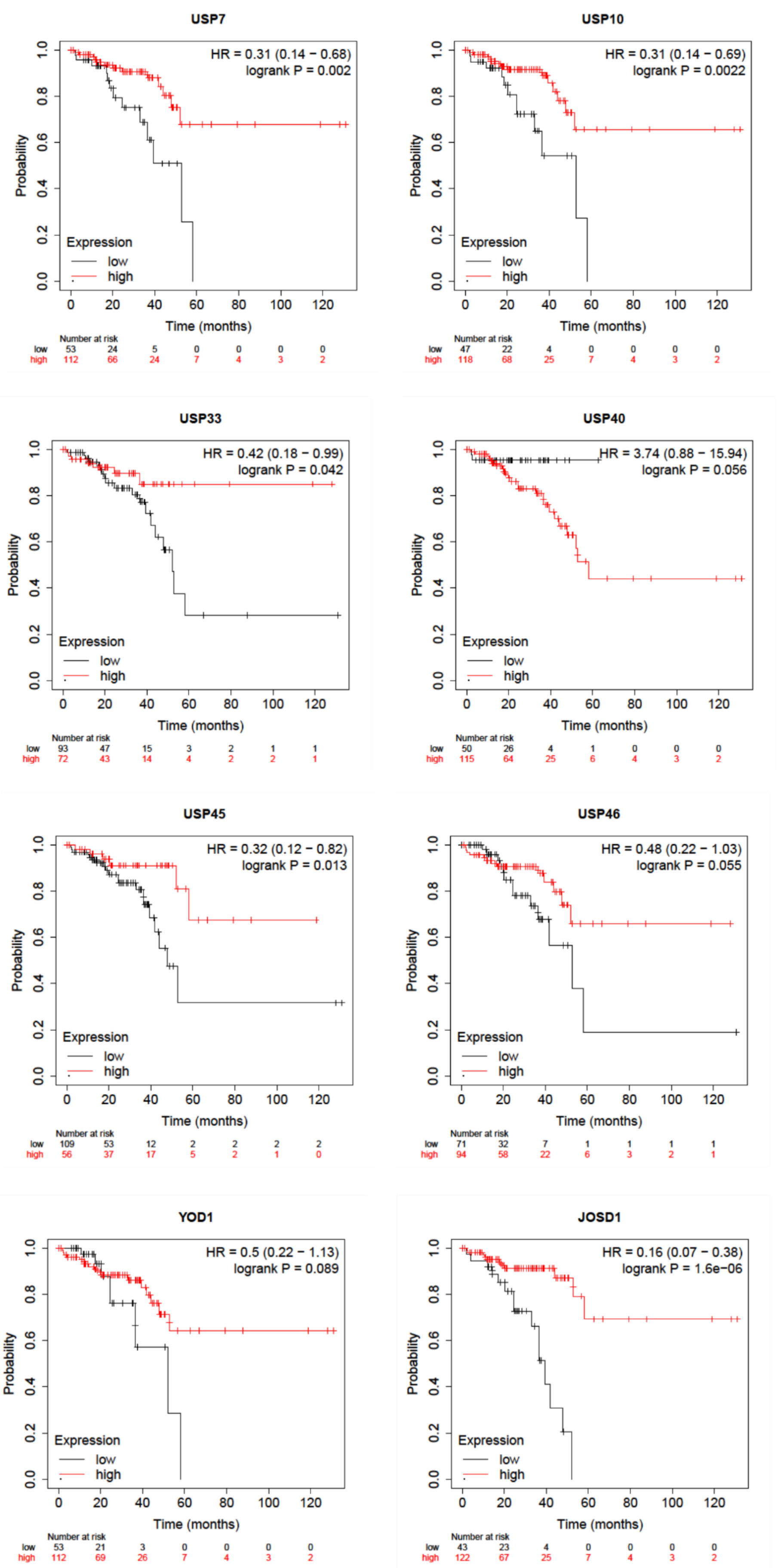

Supplementary Figure S1. Kaplan-Meier overall survival analysis of 165 colorectal adenocarcinoma patients for USP7, USP10, USP33, USP40, USP45, USP46, YOD1 and JOSD1 RNASeq data. Data were generated with the online tool LM plotter (http://kmplot.com) [43].

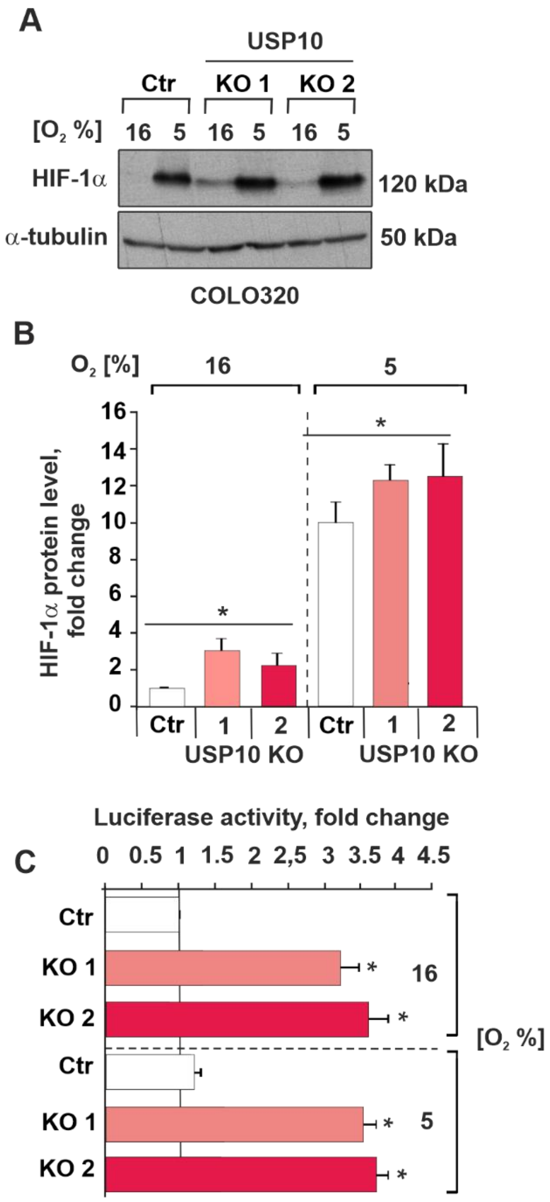

Supplementary Figure S2. USP10 affects HIF-1α protein level in COLO320 cells. (A) Representative immunoblot of USP10 and HIF-1α levels in the lysates of COLO320 Ctr cells and in USP10 KO1 and USP10 KO2 cells. Cells were cultured under normoxic (16% O_2) or hypoxic conditions (5% O_2) for 5 hours. α-tubulin served as loading control. (B) Analysis of HIF-1α protein levels in Ctr COLO320 cells was set as 1 (C) COLO320 Ctr and USP10 KO1 and KO2 cells were transfected with a Luc reporter gene construct pGL3-EPO-HRE-Luc and treated with 5% O_2 for 18 hours. The Luciferase activity of pGL3-EPO-HRE-Luc transfected Ctr at 16% O_2 was set to 1. The values are mean ± SD of 3 independent experiments; * significant difference ($p < 0.05$).

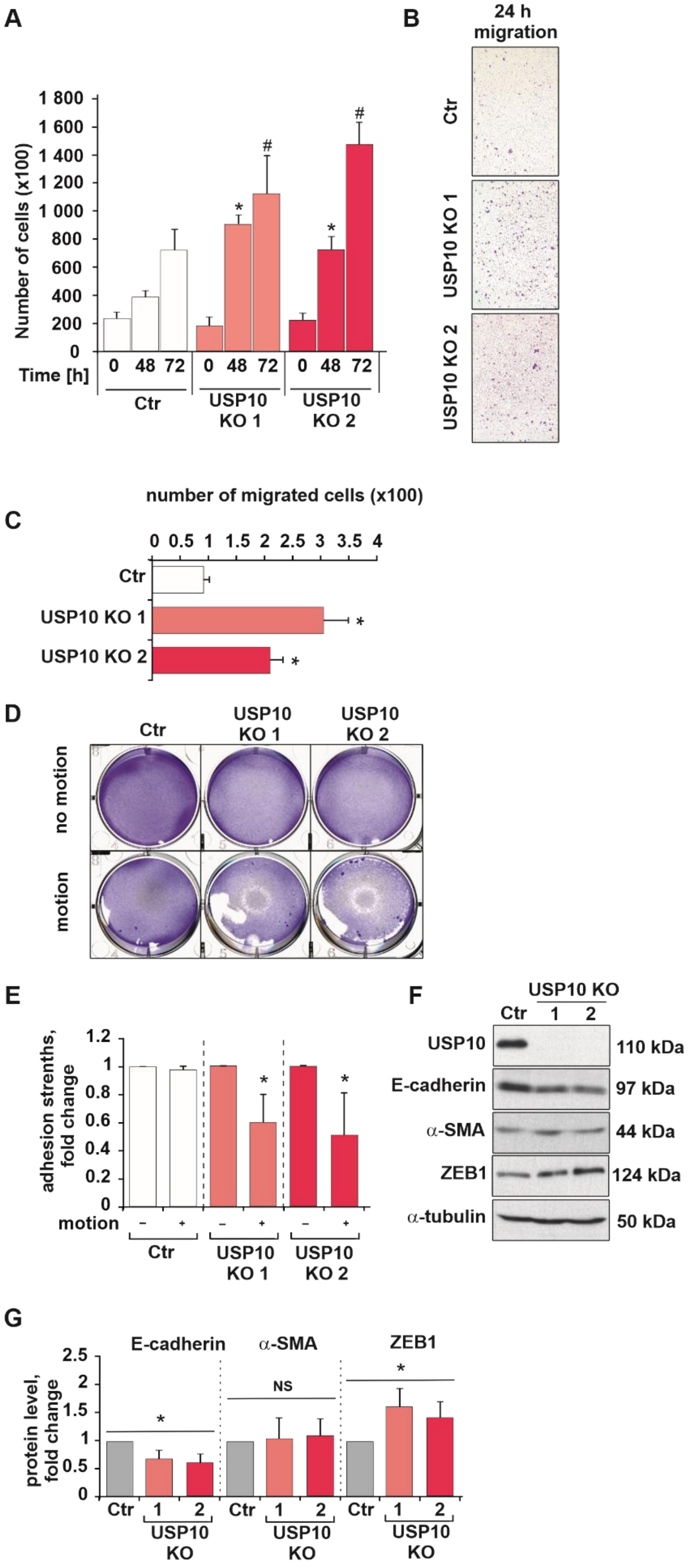

Supplementary Figure S3. USP10 knockout increases the proliferation and motility and decrease adhesiveness in HCT116 cells. (A) Analysis of cell count assay of HCT116 control cells and USP10 lacking cells measured every 24, 48 and 72 hours. (B) Representative images of the cell culture inserts from the 24 hour migration assay of HCT116 Ctr cells and USP10 KO1 and KO2 cells. (C) Quantification of the Transwell migration assay towards serum. (D) Representative images of the whole cell culture wells after adhesion assay. (E) Analysis of cellular adhesion strengths. Adhesion of motionless samples was set to 1. (F) Representative immunoblot of E-Cadherin, α-SMA and ZEB1 levels in the lysates of HCT116 Ctr cells and in USP10 KO1 and USP10 KO2 cells. α-tubulin served as loading control. (G) Analysis of E-Cadherin, α-SMA and ZEB1 protein levels in Ctr HCT116 cells was set as 1 Data are mean ± SD of 3 independent experiments; * significant difference ($p < 0.05$) between USP10 KO cells vs. Ctr.
